# New contribution to the morphology and molecular mechanism of *Euplotes encysticus* encystment

**DOI:** 10.1038/s41598-018-31160-8

**Published:** 2018-08-24

**Authors:** Fenfen Chen, Yanyan Xue, Nan Pan, Muhammad Zeeshan Bhatti, Tao Niu, Jiwu Chen

**Affiliations:** 1 School of Life Sciences, East China Normal University, Shanghai, 200241 P. R. China; 2 Institute of Biomedical Sciences, School of Life Sciences, East China Normal University, Shanghai, 200241 P. R. China; 3 Department of Molecular Medicine, National University of Medical Sciences, Rawalpindi, Pakistan

## Abstract

Ciliated protists are a large group of single-cell eukaryotes, leading to the resting cysts in unfavorable environmental condition. However, the underlying molecular mechanism of encystment in the free-living ciliates is poorly understood. Here we show that the resting cysts are better than the vegetative cells of *Euplotes encysticus* in adverse survivor with respect to energy metabolism. Therefore scale identification of encystment-related proteins in *Euplotes encysticus* was investigated by iTRAQ analysis. We analyzed a total of 130 proteins, in which 19 proteins involving 12 upregulated and 7 downregulated proteins were associated with encystment in the resting cysts in comparison with the vegetative cells. Moreover, direct fluorescent labeling analysis showed that the vegetative cells treated with shRNA-β-tubulin recombinant *E. coli* accumulated a large number of granular materials, and dramatic cell morphology changes. Importantly, the cell membrane rupture phenomenon was observed after three weeks of shRNA-β-tubulin interference as compared to the control group. These results revealed that different proteins might play an important role in the process of the vegetative cells into the resting cysts. These results will help to reveal the morphological changes and molecular mechanism of resting cyst formation of ciliates.

## Introduction

Ciliates are highly differentiated, morphological complex and widespread unicellular eukaryotes^1,2^. Ciliate encystment involves progressive and drastic physiological and morphological changes, including significant reduction of cellular volume, presence of cyst walls, cytoplasmic dehydration, and high autophagic activity^3,4^. The resting cyst formation in the life cycle of protozoa is considered as a protective mechanism against unfavorable environmental conditions, such as starvation, high population density, temperature, *etc*. The vegetative cells differentiate into resting cysts in emergence under adverse conditions, whereas excystment occurs when environmental conditions become favorable^5^. This process is called encystment or cyst formation, and considered as a superior strategy for most ciliates to survive under the stressful conditions in their environments. Basically, encystment is a relatively common phenomenon among free-living ciliates, particularly for freshwater species^6^. This process is an excellent model system to elucidate the molecular mechanism of morphogenesis at cellular level. Therefore, ciliate encystment is widely paid attention. Recently, the morphological, physiological and biochemical changes occurring during free-living ciliate encystment have been extensively studied^7,8^. However, the molecular mechanisms of free-living ciliate encysment remains largely unknown, especially regulation of protein expression during encystment.

The aim of this study was to elucidate the molecular mechanism of encystment in resting cysts. To gain insight into detail mechanism, we primarily search for proteomic profile of *Euplotes encysticus* in encystment, particularly focus on revealing the encystment-related proteins. In isobaric tags for relative and absolute quantitation (iTRAQ)-based identification and quantification of proteins between the vegetative cells and the resting cysts revealed the function and molecular mechanism of the resting cyst formation. Moreover, β-tubulin expression in the resting cyst was higher than the vegetative cells. These results will help to better understand of the adaptation and protection mechanism underlying ciliates in environmental changes and molecular mechanism of ciliate encystment.

## Results

### Energy metabolism analysis of the vegetative cells and the resting cysts

**Figure 1:**
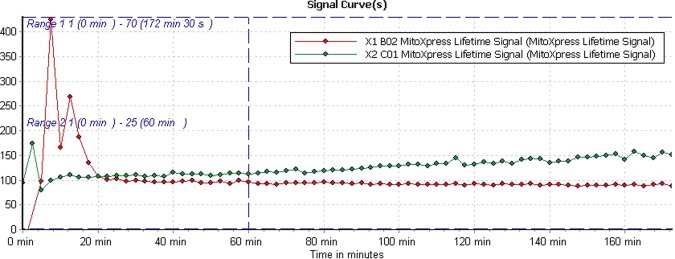
The life signal curve of the vegetative cells and the resting cysts of *Euplotes encysticus*. A red color curve represents the vegetative cells and green color shows the resting cysts.

The energy metabolism life signal curve of the vegetative cells and the resting cysts were obtained by Omega-Data analysis. We found that life signal of the vegetative cells was significantly higher than the resting cysts in the initial 20 min with adequate supply of oxygen. Conversely, life signal of the resting cysts was gradually increased after 20 min till 150 min. These variations occur due to sealed experimental condition, because the resting cysts were more adaptive to low oxygen as compared to the vegetative cells (Fig. 1). These results indicated that the resting cysts are more sustain in adverse survivor as compared to the vegetative cells.

### Electrophoresis for protein analysis

**Figure 2:**
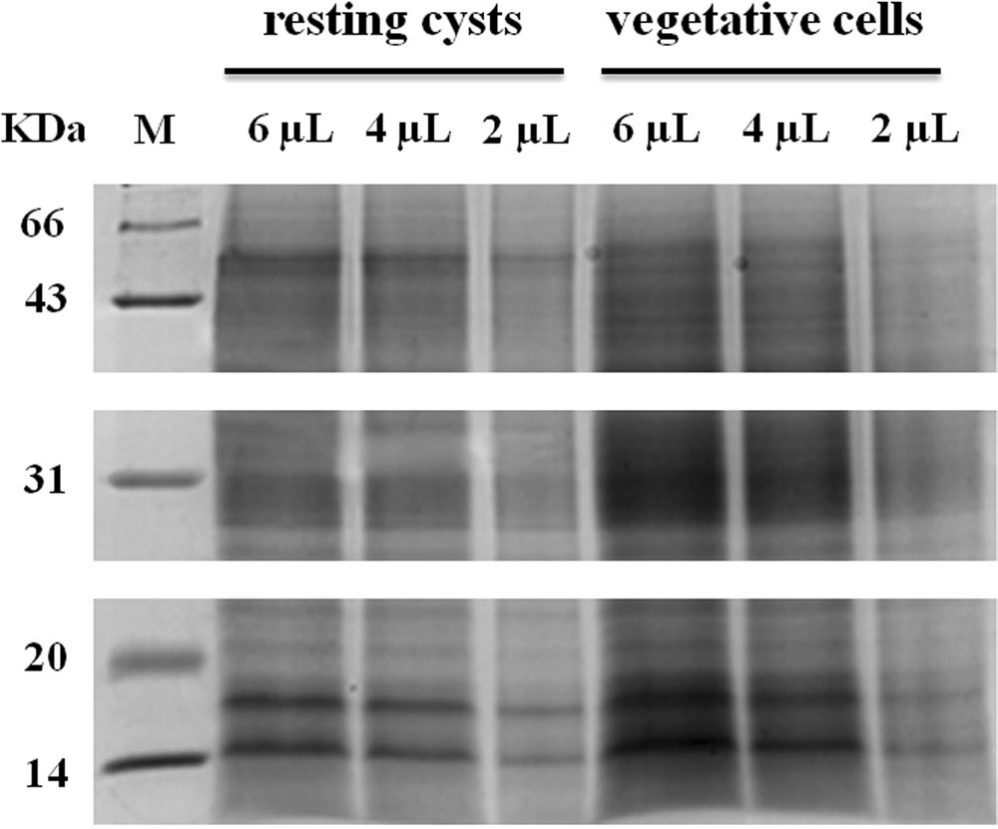
Proteins expression of the vegetative cells and the resting cysts by SDS-PAGE map. The protein was extracted from the vegetative cells and the resting cysts. The samples were separated by 12% SDS-PAGE gel and stained with Coomassie Brilliant Blue R-250. M: marker for protein molecular weight.

Our experiment on energy metabolism life signal showed that ability of the resting cysts to resist adversity was much better than that of the vegetative cells. To insight into the molecular mechanism of the resting cysts resistance to adversity, encystment-related proteins were considered. We investigated the total protein of the vegetative cells and the resting cysts by SDS-PAGE analysis. Our findings demonstrated that protein distribution bands of the vegetative cells and the resting cysts were similar, whereas the resting cysts showed lighter band comparing to the vegetative cells (Fig. 2; Full image is shown in Fig. S1). These results suggest that the two samples had good consistency, relative accuracy, and can be used for iTRAQ experiments.

### Mass spectrometry analysis

**Figure 3:**
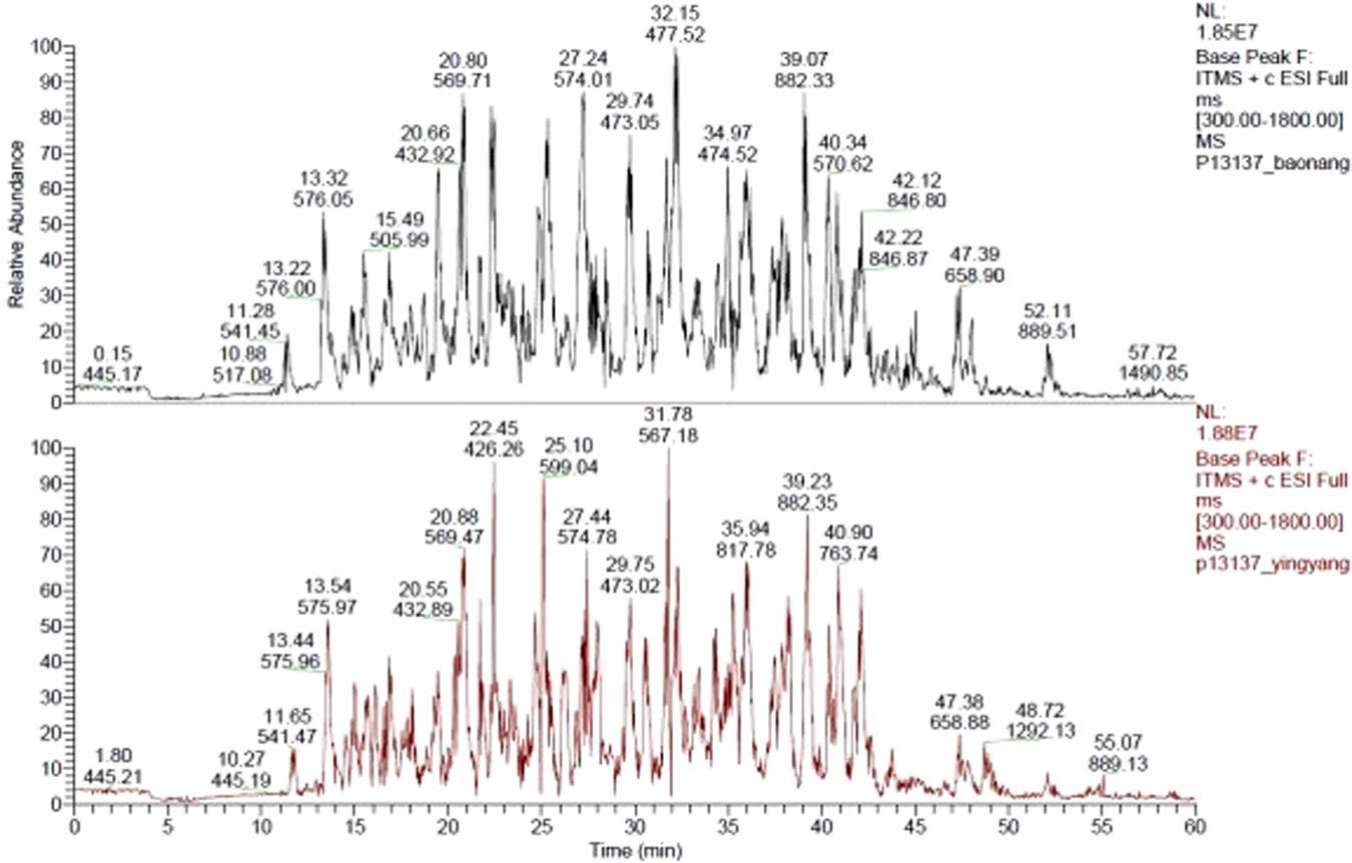
The mass spectrum basepeak maps of the samples.

Based on the proteins identification of the vegetative cells and the resting cysts *via* SDS-PAGE electrophoresis analysis. Further, investigation was carried out through mass spectrometry (MS) analysis. The results indicated that peaks of 157 resting cyst proteins and 198 proteins of vegetative cells were identified (Fig. 3). Although, some difference of the vegetative cell and the resting cyst proteins occur in MS analysis comparing to SDS-PAGE identification proteins. Furthermore, statistical analysis demonstrated that most of the proteins were shared by the vegetative cells and the resting cysts such as β-tubulin. Taken together, β-tubulin is one of the most important proteins in the vegetative cells and the resting cysts.

### Protein identification and quantification

**Figure 4:**
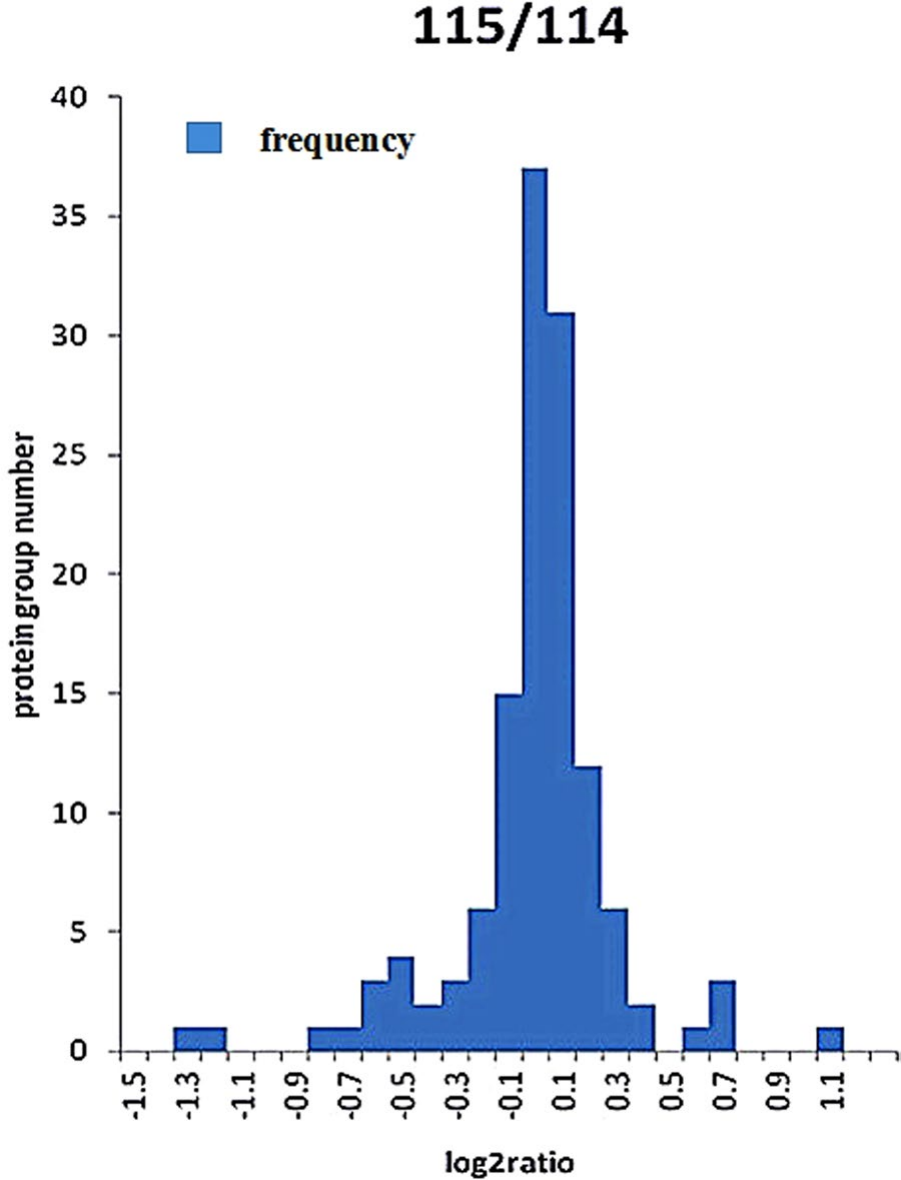
Frequency distribution histogram of quantitative ratio of the samples.

A total of 130 proteins were identified by iTRAQ-coupled LC-MS/MS quantification of differentially expressed proteins in the vegetative cells and the resting cysts of *Euplotes encysticus*. Significant up/down regulations of the vegetative cells and the resting cyst differentially expressed proteins were determined by a log2 ratio of >0.1 and *p-*value < 0.05 for 115:114 ratio. A total of 130 proteins were revealed to be differentially expressed. Among them, 19 proteins were significantly up or downregulated in the vegetative cells and the resting cysts (Fig. 4). Therefore, 19 significantly differentially expressed proteins are listed in Table 1. Specifically, 12 proteins were downregulated and 7 proteins were upregulated in the vegetative cells. Whereas, 12 proteins were upregulated and 7 proteins were downregulated during the resting cysts exposure. The results showed that β-tubulin and β-tubulin T2 are associated with cytoskeleton-related proteins. Moreover, chromosome constitute proteins such as H2A, H2B, H3, H4, while TBP-interacting protein, elongation factor 1α, elongation factor Tu, elongation factor Tu C-terminal domain protein were related to DNA replication and expression. Several proteins play a role in the energy synthesis and metabolism, such as dihydrolipoate dehydrogenase, ADP/ATP carrier, ATP synthase β subunit, isocitrate dehydrogenase, cytochrome b, S-adenosylmethionine synthase. Further, another two differentially expressed proteins were 50S Ribosomal protein L14 and heat shock chaperone (HSP-70), respectively. 50S Ribosomal protein L14 is a novel protein for ribosome, while HSP-70 involved in the correct folding of proteins and degradation of misfolded proteins.

**Table 1:** The significant different proteins in the vegetative cells and the resting cysts from *Euplotes encysticus*.

Accession No.	Name	Peptides (95%)	Unused	% Cov	Significant analysis	
					115/114	115/114 *p* value
tr|A1YZ12|A1YZ12_9CILI	Beta-tubulin (Fragment) OS = Myrionecta rubra PE = 2 SV = 1	55	2	61.2	0.3873	1.98E-09
tr|M3S210|M3S210_ENTHI	Elongation factor Tu C-terminal domain containing protein OS = Entamoeba histolytica HM-1:IMSS-B GN = EHI8A_141130 PE = 4 SV = 1	3	4.02	19	0.413	2.29E-08
tr|Q4UEX0|Q4UEX0_THEAN	Histone H2A OS = Theileria annuLata GN = TA14155 PE = 3 SV = 1	3	6.1	26.3	0.5395	0.000103
sp|O97484|H2B_EUPCR	Histone H2B OS = Euplotes crassus GN = H2B1 PE = 3 SV = 1	14	8.39	46.9	0.5754	0.000519
tr|Q868U8|Q868U8_EUPAE	Beta-/gamma-platein OS = Euplotes aedicuLatus PE = 4 SV = 1	7	6	15.2	0.631	0.003955
tr|C0L7F0|C0L7F0_EUPFO	Beta-tubulin T2 OS = Euplotes focardii PE = 3 SV = 1	68	71.07	66.9	0.6368	0.004754
tr|Q3I4X1|Q3I4X1_EUPCR	Elongation factor Tu OS = Euplotes crassus PE = 3 SV = 1	1	2	9.5	0.6427	0.005703
tr|Q8MUZ9|Q8MUZ9_EUPAE	Histone H4 OS = Euplotes aedicuLatus PE = 3 SV = 1	13	10.3	56.1	0.6668	0.01143
tr|J9INL0|J9INL0_9SPIT	DNA helicase TIP49, TBP-interacting protein OS = Oxytricha trifallax GN = OXYTRI_21730 PE = 4 SV = 1	1	2	9	0.673	0.013505
tr|Q71LX5|Q71LX5_EUPOC	Histone H3 OS = Euplotes octocarinatus PE = 3 SV = 1	4	5.95	38.2	0.7047	0.02954
tr|D7PJI8|D7PJI8_9DINO	50 S ribosomal protein L14, chloroplastic OS = Kryptoperidinium foliaceum GN = rpl14 PE = 3 SV = 1	1	2	17.4	0.7047	0.02954
tr|Q0WY77|Q0WY77_9APIC	Heat shock protein 70 (Fragment) OS = Theileria ovis GN = hsp70 PE = 3 SV = 1	3	2	17.6	0.7178	0.039536
tr|J9I0E6|J9I0E6_9SPIT	Dihydrolipoyl dehydrogenase OS = Oxytricha trifallax GN = OXYTRI_06149 PE = 3 SV = 1	1	2	17.4	1.1912	0.045524
tr|Q0VUC6|Q0VUC6_STYLE	Isocitrate dehydrogenase [NADP] (Fragment) OS = Stylonychia lemnae GN = idh PE = 3 SV = 1	2	4.01	25.6	1.2246	0.021449
tr|Q5BLW9|Q5BLW9_9SPIT	Cytochrome b OS = Pseudourostyla cristata GN = CYTB PE = 4 SV = 2	1	2	3.6	1.2706	0.006927
tr|O96975|O96975_EUPAE	Elongation factor 1-alpha (Fragment) OS = Euplotes aedicuLatus GN = TEF1 PE = 3 SV = 1	17	19.74	33.4	1.3062	0.002697
tr|J9J9J2|J9J9J2_9SPIT	ATP synthase subunit beta OS = Oxytricha trifallax GN = OXYTRI_17619 PE = 3 SV = 1	12	14.46	30.7	1.4723	1.71E-05
tr|Q8MVR4|Q8MVR4_9SPIT	ADP/ATP carrier OS = Euplotes sp. GN = AAC PE = 3 SV = 1	3	2	20.6	1.5417	1.59E-06
tr|J9F342|J9F342_9SPIT	S-adenosylmethionine synthase OS = Oxytricha trifallax GN = OXYTRI_06221 PE = 3 SV = 1	1	2	7.9	1.5704	5.73E-07

Besides these specific proteins in 115:114, some other identified proteins with apparently altered abundance without 115:114 rations are listed in Table 2. Overall, 6 proteins were identified, such as adenosylhomocysteine, glyceraldehyde 3-phosphate-dehydrogenase, serine/threonine protein phosphatase, the vacuolar ATP synthase catalytic subunit, and the mitotic cycle protein 48 homologue in the vegetative cells. These different proteins were thought to be involved in a broad range of function including conversion process of the vegetative cells to the resting cysts.

**Table 2:** The proteins without 115/114 ratio in the vegetative cells or the resting cysts from *Euplotes encysticus*.

Accession No.	Name	Peptides (95%)	Unused	% Cov
tr|C5LXA1|C5LXA1_PERM5	Putative uncharacterized protein OS = Perkinsus marinus (strain ATCC 50983 / TXsc) GN = Pmar_PMAR016893 PE = 3 SV = 1	1	2	10.8
tr|F0VBY5|F0VBY5_NEOCL	Glyceraldehyde 3-phosphate dehydrogenase, related OS = Neospora caninum (strain Liverpool) GN = NCLIV_041940 PE = 3 SV = 1	2	2	13.8
tr|J9II79|J9II79_9SPIT	Serine/threonine-protein phosphatase OS = Oxytricha trifallax GN = OXYTRI_24298 PE = 3 SV = 1	2	2	16.7
tr|Q7RII4|Q7RII4_PLAYO	Cell division cycle protein 48 homolog OS = Plasmodium yoelii yoelii GN = PY03639 PE = 4 SV = 1	4	2.03	12.9
tr|G0R4D6|G0R4D6_ICHMG	Adenosylhomocysteinase OS = Ichthyophthirius multifiliis (strain G5) GN = IMG5_191490 PE = 3 SV = 1	2	4.22	12.6
tr|G0R4P2|G0R4P2_ICHMG	Vacuolar ATP synthase catalytic subunit a, putative OS = IchthyopHthirius muLtifiliis (strain G5) GN = IMG5_194110 PE = 3 SV = 1	6	10.07	18.8

### Morphological changes of *Euplotes encysticus* by FLUTAX fluorescent labeling analysis

**Figure 5:**
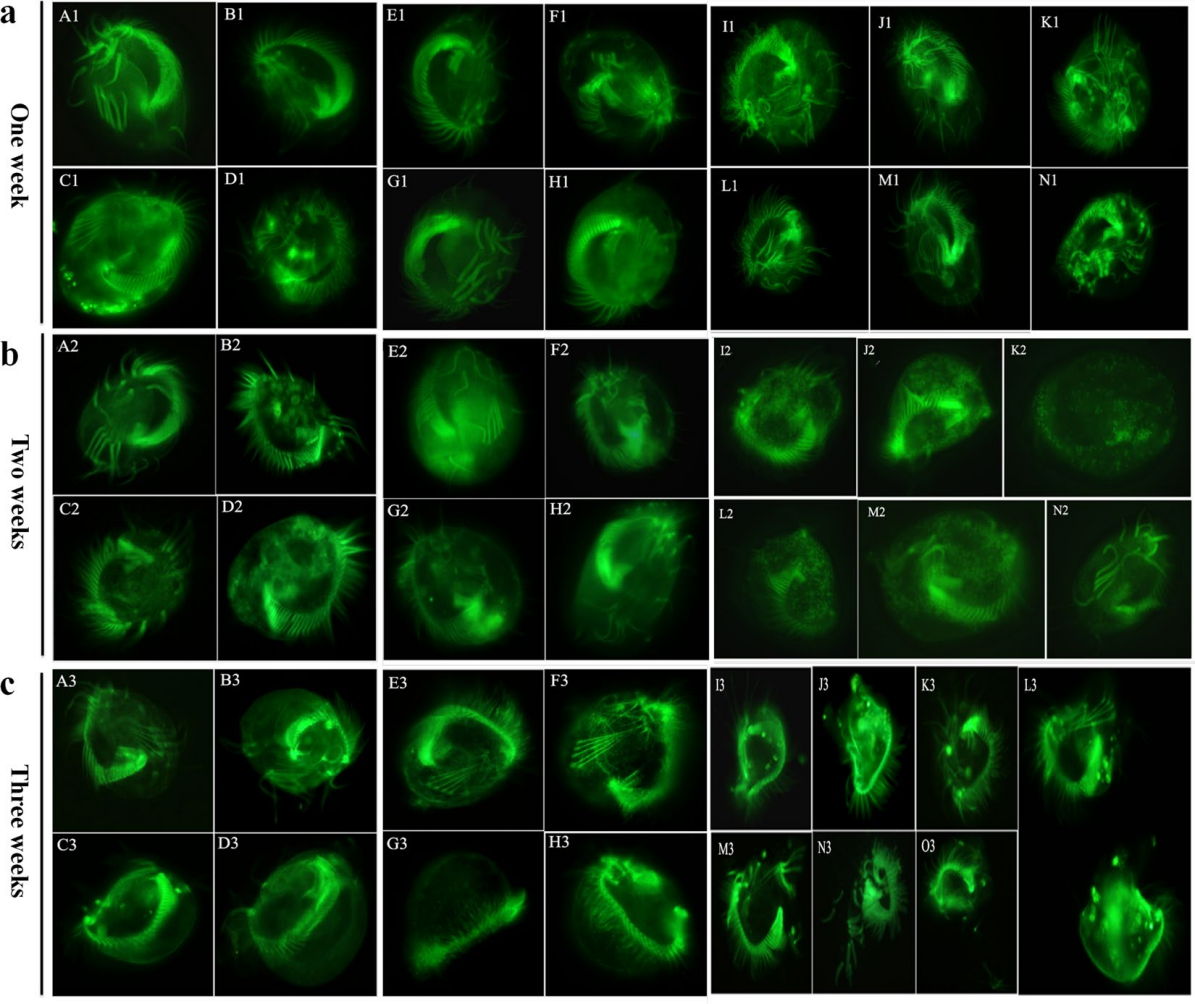
The morphological changes of *Euplotes encysticus* by RNA interference. The vegetative cells were feed with or without shRNA-β-tubulin interference for 1–3 weeks. The representative images of the vegetative cells (**a**) A1-D1: blank, E1-H1: negative control, I1-N1: after one week of shRNA-β-tubulin interference. (**b**) A2-D2: blank, E2-H2: negative control, I2-N2: after two weeks of shRNA-β-tubulin interference. (**c**) A3-D3: blank, E3-H3: negative control, I3-N3: after three weeks of shRNA-β-tubulin interference. The wheat fermentation broth fed to the vegetative cells was used as blank and the vegetative cells fed with *E. coli* serve as negative control. Images were taken photos by Olympus BX51 fluorescence microscope (excitation wavelength = 492 nm, emission wavelength = 520 nm) at 40× magnification.

The β-tubulin plays an important role in the morphology of ciliates and changes of cytoskeleton. Through the statistical analysis, we found that β-tubulin protein was significantly upregulated in the encystment. Therefore, FLUTAX fluorescent labeling analysis was performed after one week of shRNA-β-tubulin interference. Results indicated that no dramatic changes were obtained in the oral girth, frontal spine hair, transverse spine hair, and tail spin hair (Fig. 5a). However, slight differences in the green fluorescence emission by round particles were recorded in the shRNA-β-tubulin interference group comparing with control group (Fig. 5a, K1-N1). These results suggesting that one week duration was not enough for morphological structural changes. Therefore, we extended the interference period of shRNA-β-tubulin interference (Fig. 5b) to two weeks. We found that large number of particles with green fluorescence was appeared in the cytoplasm of the shRNA-β-tubulin interference group (Fig. 5b, I2-N2). Conversely, no significant changes were noted in the basic structure of the shRNA-β-tubulin interference group (Fig. 5b, I2-N2). These basic structures were similar to the basic structure of blank and negative control group (Fig. 5b, A2-H2). To further gain insight into the morphological changes, we aimed to detect the cell morphological changes after three weeks in the presence of shRNA-β-tubulin interference. Our findings indicated that cell shape and cell volume was dramatically changed in shRNA-β-tubulin interference group. Moreover, cell rupture and cell contents spillage was exhibited by the shRNA-β-tubulin interference group comparing with control group (Fig. 5c, I3-K3). Besides, cell contents overflow from the anterior longitudinal microtubules of the cells (Fig. 5c, L3, M3), and part of cilia structure was fallen off in the shRNA-β-tubulin interference group (Fig. 5c, N3, O3). Whereas, cell morphology and cell volume was similar in blank and negative control group (Fig. 5c, A3-H3). Taken together, the results showed that after 3 weeks shRNA-β-tubulin interference affected the microtubules in the cortex, and certain influence on the cytoskeleton. Moreover, cells sharking and diverse morphologies were obtained in the shRNA-β-tubulin interference group. Additionally, Fig. 5c suggested that resting cysts were difficult to form under β-tubulin gene interference.

## Discussion

*Euplotes encysticus* is an aerobic organism, accompanied with energy metabolism in the aerobic respiration process to adapt the environment survive. Numerous reports have focused on the morphology of cysts^6,8^. Previously, we reported cyst wall proteins function and molecular mechanism of *Euplotes encysticus* under stress condition^9^. In this article, the life signal curve of the vegetative cells and the resting cysts from *Euplotes encysticus* was obtained by using the energy metabolism analysis. As shown in Fig. 1, during the first 20 min of the aerobic period, the vegetative cell metabolism was higher than the resting cyst. Subsequently, the metabolism of the vegetative cells rapidly declined and entered a state of death due to lack of oxygen. In contrast, resting cysts showed survival in low metabolic levels after 20 min which demonstrates their ability to resist adverse conditions.

Ciliates encystment is a RNA and protein synthesis dependent process^4,10^. The resting cysts and the vegetative cells have specific proteins. These proteins distribution were identified by SDS-PAGE electrophoresis with dose-depend manner. Furthermore, iTRAQ-based proteomic analysis was performed to identify the 130 differential expression proteins between the vegetative cells and the resting cysts of *Euplotes encysticus*. Among them, 19 proteins exhibited significant differentiation. As compared with the vegetative cells, 12 upregulated and 7 downregulated proteins were expressed in the resting cysts. Likewise, 6 specific proteins in the vegetative cells or the resting cysts were identified without 115:114 ratios. These proteins are widely distributed and involved in various life activities of the cells, including the transport of substances and energy metabolism, expression and replication of genetic material, stress response, and signal transduction. These proteins involved in the conversion of the vegetative cells to the resting cysts during adverse effect of environment. When the environment is conducive to survive, the resting cysts convert into the vegetative cells. The analyses of encystment-related proteins were screened by comparing the protein expression difference between the vegetative cells and the resting cysts, which could reveal the molecular mechanism of ciliate encystment at the protein level. We got a total of 19 differential expression proteins. The upregulation proteins in the resting cysts includes β-tubulin, β-tubulin T2, histone H2A, histone H2B, histone H3, histone H4, DNA helicase TIP49, 50S ribosomal protein L14, heat shock protein 70, elongation factor Tu. The downregulated proteins such as dihydrolipoyl dehydrogenase, isocitrate dehydrogenase, S-adenosylmethionine synthase, ADP/ATP carrier, ATP synthase subunit beta, cytochrome b, and elongation factor 1-alpha. With respect to the upregulated proteins, β-tubulin is an important component in the cytoskeleton and highly expressed. Our iTRAQ results showed that the β-tubulin expression was significantly higher, which plays an important role in the encystment process.

Previous studies demonstrated that morphological structure of the macronucleus in *Euplotes encysticus* changed significantly during the encystment, and the chromatin loosely distributed and honeycombed^11^. However, no difference was observed in the micronucleus before and/or after encystations formation. Similarly, our findings indicated that the expression of histones which are components of chromosome had undergone significant changes during the encystment. The expression of various histones (H2A, H2B, H3, and H4) in the resting cysts was obviously enhanced as compared to the vegetative cells. It is well known that micronucleus in ciliates generally control the heredity of cells, macronucleus control of cell nutrition. The upregulation expressions of these histones were consistent with the change of the macronucleus morphological structure of the resting cysts. Some evidences have been reported that histones sharply change cell structure and morphology, *e.g*. histones (H2A, H2B, H3, H4) high expression leads to the activation of chromatin remodelers^12^. Our data showed upregulated expression of histones (H2A, H2B, H3, and H4), which is in accordance with the previous studies.

Under adverse circumstances, ciliates themselves will produce complex biological responses to the perception of stress signals, regulate the expression of stress-related proteins in the cells and then form cysts to withstand adverse conditions. Our results showed that HSP70 and elongation factor Tu expression was significantly increased in the resting cysts. HSP70 belongs to the chaperone protein family whose expression is induced by different stresses. In response to the extracellular or intrinsic stimuli and stresses, HSP70 plays important roles in the maintenance of cellular homeostasis and cellular defense system to minimize damages from cellular stresses^13^. Elongation factor Tu is an important elongation factor, which plays role in the transportation of aminoacyl-tRNA complexes during translation and stress response^14^. Therefore, HSP70 and elongation factor Tu expression upregulation could strengthen the encystment for resisting against adverse conditions and these genes expression must markedly changed. Our results indicated that enhanced expression of DNA helicase TIP49, and 50S ribosomal protein L14 was observed. DNA helicase TIP49 possesses multiple sequence motifs for ATPase and DNA helicase. It is involved in wide range of cellular processes including DNA damage response and cellular transformation. DNA helicase TIP49 is also a part of various chromatin remodeling complexes and key component for their activities^15–18^. 50S ribosomal protein L14 is a protein of the 50S ribosomal subunit. Its function is to bind to 23S rRNA and form part of two inter-subunit bridges in the 70S ribosome^19^. 50S ribosomal is a macromolecular complex, which participates in protein translation and 50S ribosomal protein L14 participates in the encystment-related proteins translation. Therefore, 50S ribosomal protein L14 and DNA helicase TIP49 possibly play important roles in the encystment.

Among the downregulated proteins, ATP synthase subunit beta and ADP/ATP carrier are vital proteins for oxidative phosphorylation in the mitochondria of eukaryotic cells. ATP synthase is critical in defining the bioenergetic activity of the cell and synthesizes ATP from ADP and phosphate. The β-subunit of the mitochondrial ATP synthase catalyzes the rate-limiting step of ATP formation in eukaryotic cells^18^. Whereas, the key transport steps for oxidative phosphorylation in eukaryotic organisms are the mitochondrial ADP/ATP carriage, which import ADP from the cytosol and export ATP from the mitochondrial matrix^20^. The transportation processes use a specialized transporter proteins ADP/ATP carrier to export the mitochondria ATP to the cytoplasm which can provide energy to the cells^21^. Cytochrome b is an important protein of eukaryotic cells in the mitochondria, which plays role in the function of electron transport chain and acts as a main subunit of transmembrane cytochrome bc1 and b6f complexes^22,23^. These complexes are involved in electron transport to pump of protons to create a proton-motive force. This proton gradient is used for the generation of ATP^24^. Our results indicated that cytochrome b, ADP/ATP carrier and ATP synthase subunit beta expression was significantly downregulated in the encystment process, which suggested that energy metabolism in the resting cysts was lower than the vegetative cells. These results are consistent with our recent data from energy metabolism analysis.

iTRAQ analysis indicates that some significantly downregulated proteins are enzymes, which were associated with substance and energy metabolism. These enzymes expression level in the resting cysts was significantly lower than the vegetative cells, which is consistent with the characteristics of the resting cyst with low substances and energy metabolism. Thus, the expression of enzymes related to energy metabolism in the resting cysts should be lower than the vegetative cells. Among these enzymes, S-adenosylmethionine synthetase (SAMS) is an enzyme that synthesizes S-adenosylmethionine (SAM) using methionine and ATP. SAMS is methyl donor in intracellular methylation pathway of cell metabolism. SAM in the metabolic process involves a lot of enzymes, these enzymes have wide range of biological functions, including modification of proteins and nucleic acids, and some of the basic metabolism^25,26^.

It is well known that tricarboxylic acid (TCA) cycle reaction is catalyzed by the pyruvate dehydrogenase complex containing pyruvate dehydrogenase, dihydrolipoyl transacetylase and dihydrolipoyl dehydrogenase. Isocitrate dehydrogenase is responsible for catalyzing the oxidative decarboxylation of isocitrate to α-ketoglutarate in the TCA cycle, and reduces NDA^+^ to NADH. Since isocitrate dehydrogenase is the rate-limiting enzyme of the TCA cycle, isocitrate dehydrogenase has great influence on the metabolism of organism. In our study, we found that SAMS, dihydrolipoyl dehydrogenase and isocitrate dehydrogenase in the resting cysts were significantly lower, suggesting the resting cysts only maintaining some of the basic metabolic activities to survive. Further, EF-1α was downregulated in the resting cysts which suggested that synthesis of the corresponding proteins decreased, whereas EF-Tu expression upregulated. The expression level between these two elongation factors in the resting cysts was opposite, mainly because of the different roles of both in the resting cysts. EF-1α is a highly modified protein, but not EF-Tu. The role of EF-1α in protein synthesis is similar to that of EF-Tu. EF-1α is also involved in many other cellular processes such as proteasomal degradation, and nuclear export^27^. So EF-1α expression downregulation hinted these cellular processes decreased. This supported the cyst dormant state *Euplotes encysticus* transformed the vegetative cells into the resting cysts should also express some specific proteins. Table 2 listed some proteins without 115:114 ratio, they may be specific proteins in the vegetative cells or the resting cysts.

Obviously, the above encystment-related proteins are involved in various cell life activities, co-guide encystment, namely, promoted the conversion of the vegetative cells to the resting cysts upon encountering adversity. The discussion of these encystment-related proteins functions will shed light on some encystment-related questions. In order to further study the function of the encystment-related proteins, we selected the β-tubulin from the apparently upregulated proteins for morphological variations. We used shRNA combined with FLUTAX fluorescence labeling technology to detect morphological changes of the vegetative cells with shRNA-β-tubulin interference. We found that the cell morphology was changed dramatically by the vegetative cells upon transferring into the resting cysts, which suggested that the cytoskeleton also undergoes drastic changes. Moreover, the external and internal morphologies of *Euplotes encysticus* have been changed with shRNA interference. There was accumulation of round particulate matter in the vegetative cells at 1^st^ and 2^nd^ weeks of treatment. After the 3^rd^ week of interference, the cell morphology was significantly changed and the cell volume became smaller. A variety of malformations occurred and the cells were easily ruptured, the cytoplasmic spillover, the transaxial spine capillary leakage, and some external cilia structure shedding. In this study, we showed that β-tubulin gene interference has effective influence on *Euplotes encysticus*. The morphology of *Euplotes encysticus* was significantly changed after interfering β-tubulin gene, the interference of β-tubulin leading to the blockage of β-tubulin expression, and in turn interfere the structure of cytoskeletal consisting of tubulin in the cell. The intracellular or extracellular microtubules are structurally unstable and can’t maintain the normal body shape of the cells. Hong *et al*.^28^ studied the interference of the γ-tubulin gene of *E. opuntiae* and showed that the cross-sectional structure of the ciliary rod inside the cortex of the parasite after the interference became 9 + 0 and some of the cilia stems swelled. Distortion occurs and disintegration occurs, and the originally arranged microtubule structure disappears. The α, β, γ-tubulin are the basic proteins that constitute the cytoskeleton. After interfering with γ-tubulin, the microtubule structure disappears, while interference with β-tubulin gene resultant in the significant effect on the tubulin-associated structure of the cells. Thus, β-tubulin plays an essential role for cell morphological and physiological changes during encystment. These results indicated that the β-tubulin plays an important role in the cell morphology, movement, and material transportation.

## Conclusions

This study identified the energy metabolism curve between the resting cysts and the vegetative cells. One mystery underlying the encystment is the proteins expression changes. The most striking results of this study were to identify the differential expressed proteins in the resting cyst and the vegetative cells. iTRAQ-based quantification of differentially expressed proteins in the vegetative cells and the resting cysts of *Euplotes encysticus* revealing the function and interaction between eukaryocyte and environment, which insight into the morphology and molecular mechanism of resisting cysts in adverse circumstance. The results and analysis in this study will help to unravel the morphology and molecular mechanisms underlying vegetative cell into resting cysts.

## Materials and Methods

### Culture method

Hypotrich ciliate *Euplotes encysticus* was cultured in 10-cm dishes with sterilized pond water and filtered culture medium supplied by East China Normal University. After one week incubation at 25 °C with fed supply, the vegetative cells were reached at high density. Then, the vegetative cells were collected in 50 mL of vertical bottom centrifuge tubes and a total of 100 tubes were collected and subjected to starvation for 2 weeks. Then, the resting cysts and vegetative cells were washed in 1 mM Tris-HCl, pH 7.2, filtered through filter paper. The cysts and the vegetative cells on the filter paper were suspended in 1.5 mL tubes with 300 µL lysis buffer (1% SDS, 1 mM DTT, 150 mM Tris-HCL, pH 8.0; containing protease inhibitors), respectively. Then, the suspension of the resting cysts and the vegetative cells were centrifuged at 5000 rpm for 8 min and precipitation was discarded.

### Energy metabolism analysis

Briefly, an equal amount of the vegetative cells and the resting cysts were collected with an equal volume of culture water. The vegetative cells and the resting cysts were centrifuged at room temperature. Then, pellets were suspended in preheated culture water, and 150 μL of the resting cyst and the vegetative cell suspensions were added in a 96-well plates, respectively. Followed with 10 μL of probe, and 2 drops of mineral oil was added to avoid bubble formation in the mixture at 25 °C. The optimal density was determined at excitation wavelength of 380 nm and an emission wavelength of 650 nm using a micro-plate reader (Biotek, Winooski, VT, USA). The data was analyzed using the data analysis software Omega-Data analyzer.

### The sample protein extraction

The resting cysts and the vegetative cells were washed twice and collected by centrifugation and mixed with 300 µL of cell lysis buffer (4% SDS, 1 mM DTT, and 150 mM Tris-HCl, pH 8; protease inhibitors). The vegetative cells suspension was vigorously shake for 1 min and the cell layste was collected. Whereas, the cysts suspension was maintain on ice and subjected to the sonication treatment for 10 times, each time for 10 sec with a 20 sec interval, ultrasound power is 100 W. Next, the cyst lysate and the vegetative cells lysate were ultrasonic cracked in ice bath (ultrasonic 5 sec, stop 10 sec, 20 times, 100 W) and kept in boiling water for 15 min. The samples were centrifuged at 14000 × g for 15 min. The supernatants were collected and stored at −80 °C for further analysis.

### SDS-PAGE electrophoresis

Two sample protein extracts were diluted with 5× loading buffer at 5:1 (v/v), boiled for 5 min and centrifuged at 14000 × g for 10 min and then an equal volume was loaded for separation of protein to SDS-PAGE on 12% acrylamide separating gel and 5% acrylamide stacking gel at 80 V for 120 min. The prestained protein markers were run in parallel. After electrophoresis, the gels were stained with 0.25% Coomassie brilliant blue-R250 for 30 min and bleached was elute with 75 mL acetic acid, 50 mL methanol, 875 mL ddH_2_O for overnight. The molecular weight of the resting cysts and the vegetative cells were determined by molecular weights.

### Protein enzymatic hydrolysis and peptide quantification

Equal amount of the sample protein extracts were added to 100 mM DTT (Bio-Rad) and subjected to water bath at 90 °C for 5 min, and then cool to room temperature. Followed by the addition of 200 μL UA buffer (8 M urea, 150 mM Tris-HCl, pH 8.0) to the sample and mix. The sample was transferred into a 30kd ultrafiltration centrifuge tube and centrifuged at 14000 × g for 15 min. Then added 200 μL UA buffer to the sample and centrifuged at 14000 × g for 15 min. The supernatant obtained was mixed with 100 μL of iodoactamide (50 mM iodoactamide in UA buffer) and shaken (600 rpm) for 1 min. Then samples were incubated at room temperature for 30 min in dark. Next, 100 μL of UA buffer was added to the sample and centrifuged at 14000 × g for 10 min and repeated this process twice. Subsequently, 100 μL of dissolution buffer (AB SCIEX) was added to the sample and centrifuged at 14000 × g for 10 min, and repeated this process twice. The supernatant was discarded and precipitate was re-dissolved in 40 μL of trypsin buffer (3 μg Trypsin in 40 μL dissolution buffer), shaking at 600 rpm for 1 min followed by enzymatic hydrolysis at 37 °C for 16–18 h. The enzymatic hydrolysis peptides were collected by centrifuged at 14000 × g for 10 min. Finally, Optimal density of the enzymatic hydrolysis peptides were determined at a wavelength of 280 nm using a micro-plate reader (Biotek, Winooski, VT, USA).

### iTRAQ labelling

A total of 100 µg of the enzymatic hydrolysis peptides per tested sample were used for iTRAQ labeling. The samples were labeled with reagents and distributed into different groups (Applied Biosystems, Foster City, CA) according to the iTRAQ kit manufacturer’s instructions (AB SCIEX, Forster City, CA). First, we labeled the resting cyst sample with iTRAQ reagent 114, and vegetative cell sample with iTRAQ reagent 115. Then, we mixed both the samples and incubated for 1 h at room temperature. The individual sample mixed interfering substances was removed using a cation-exchange cartridge system (AB SCIXEN) according to the manufacturer’s instructions. The ion-exchange elute was desalted using C18 Cartridge (Sigma) and lyophilized. Finally, SCX fractions were analyzed using LC MS/MS system and data analysis was performed by ProteinPilot software.

### Mass spectrometry analysis

The lyophilized SCX fractions were dissolved in 40 μL of 0.1% aqueous formic acid. Then 10 μL SCX fractions were analyzed by nano-LC-MS/MS. The liquid phase system was Tempo^TM^ nanoLC (Dublin, CA, USA). The mobile-phases elution using 2% acetonitrile containing 0.1% formic acid and 98% water (mobile phase A), and 98% acetonitrile conaining 0.1% formic acid and 2% water (mobile phase B). The samples were eluted at a flow rate of 2 μL/min, enriched on a ChromXP C18 precolumn (3 μm, C18-CL, 120 Å, 360 μm × 0.5 mm). Then, the samples were separated on a Thermo scientific EASY column (75 μm × 100 mm 3 μm-C18) by 240 minute gradient at a flow rate of 300 mL/min. The gradient used is: 0–220 min, B phase rises linearly from 0 to 50%; 220–225 min, B phase rises linearly from 50% to 100%; 225–240 min B phase remains at 100%. The peptides were separated by liquid chromatography and detected by mass spectrometry (Tripple TOF^TM^ 5600 mass spectrometer of AB SCIEX). The tandem mass spectrometry detection uses Information Dependent Acquisition (IDA) mode. TOF MS scan resolution is 30,000 (FWHM), mass-to-charge ratio range is set to m/z 350–1250, cumulative time is 250 ms. The 30 most abundant peptides with a peak height over 120 cps (counts/second) and a charge of +2 to +5 were selected for MS/MS analysis and mass-to-charge ratios ranged from m/z 100 to 1800. Each TOF MS/MS scan has a cumulative time of 100 ms and a dynamic exclusion time of 18 sec. When MS/MS is acquired, the enhance iTRAQ fragmentation (Enhance iTRAQ) and automatic computing collision energy (AutoCE) functions are enabled. The data of MS analysis was analyzed using ProteinPilot Software 4.2. The ProteinPilot filter parameter: Protein FDR (False Discovery Rate) ≤0.01.

### Construction of shRNA-tubulin recombinant bacteria

Three different kinds of shRNA-tubulin recombinant plasmids were designed using the pHBAd-U6-CMV plasmid and constructed by Shanghai Sunny Biotechnology Co. Ltd. The shRNA-tubulin recombinant plasmid was mixed with *E. coli* DH5α, placed on ice for 30 min, heat shocked for 60 sec at 40 °C in a water bath, follow with cooling on ice for 2 min. The recombinant bacteria were transferred to the cultured plates containing antibiotic resistance (Ampicllin) and incubated overnight at 37 °C. The single colony of bacteria were inoculated into LB liquid medium at a dilution of 4:100 and incubated for 14 h at 37 °C with continuous shaking at 250 rpm. After extraction, shRNA-tubulin plasmid was sent to Shanghai Sunny Biotechnology Co., Ltd. for sequencing analysis. The results of sequencing are shown in Fig. S2. During the process of shRNA-tubulin induction of vegetative cells were rinsed three times with sterilized culture water and resuspended with culture water to A600 = 4.5. Then, fresh culture recombinant bacteria were fed to the vegetative cells^29^.

### FLUTAX fluorescent labeling method for revealing microtubular cytoskeleton

Fluorescent labeling was performed according to our previous method^9^ with slight modification. In brief, 40 µL of the vegetative cells were added on a clean glass slide and excess water was removed, respectively. After slides formation, 0.05% of saponin was added drop-wise and the vegetative cells were incubated for 30 sec and washed twice with PHEM. Then, the vegetative cells were fixed with paraformaldehyde 4% for 1 min and washed again with PHEM. Subsequently, treated with 0.5% Triton-X100 for 4 min and washed twice with PHEM. Finally, the vegetative cells were stained with 100 µL (1 mmol/L) FLUTAX (Invitrogen) for 8 min in dark, and washed three times with PBS (0.01 M, pH 7.2). The representative images of the vegetative cells were taken photos by Olympus BX51 fluorescence microscope (excitation wavelength = 492 nm, emission wavelength = 520 nm) at 40x magnification.

## Electronic supplementary material

Supplementary data
