# Supplementary material for: New contribution to the morphology and molecular mechanism of Euplotes encysticus encystment
Source: Sci Rep. 2018 Aug 24;8:12795. doi: 10.1038/s41598-018-31160-8 (PMC6109176; doi:10.1038/s41598-018-31160-8)
Supplement: Supplementary file 1 — Supplementary data [file 41598_2018_31160_MOESM1_ESM.docx]

**New contribution to the morphology and molecular mechanism of *Euplotes encysticus* encystment**

Fenfen Chen^1*^, Yanyan Xue^1*^, Nan Pan^1*^, Muhammad Zeeshan Bhatti^2^, Tao Niu^1^, Jiwu Chen^1**^

1. School of Life Sciences, East China Normal University, Shanghai, 200241, P. R. China

2. Institute of Biomedical Sciences, School of Life Sciences, East China Normal University, Shanghai 200241, P. R. China.

Correspondence and requests for materials should be addressed to C.J (email: jwchen@bio.ecnu.edu.cn)

*These authors contributed equally to this work.

**Correspondence author


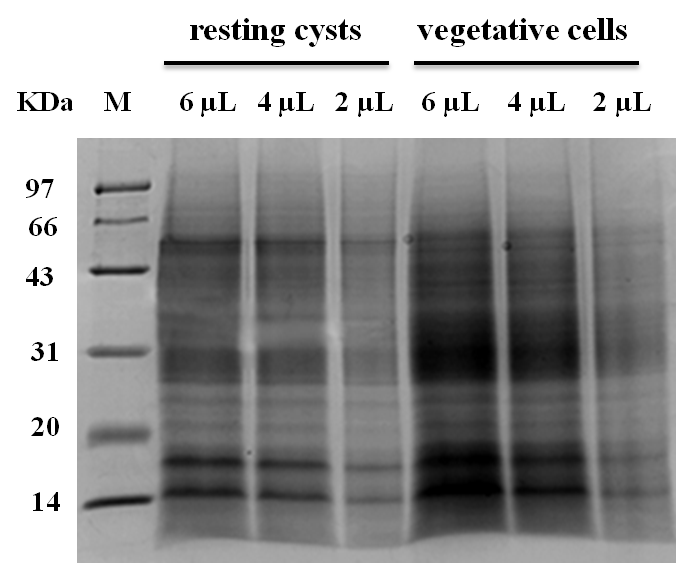


# Fig. S1. Proteins expression of the vegetative cells and the resting cysts by SDS-PAGE map. The protein was extracted from the vegetative cells and the resting cysts. The samples were separated by 12% SDS-PAGE gel and stained with Coomassie Brilliant Blue R-250. M: marker for protein molecular weight.

**
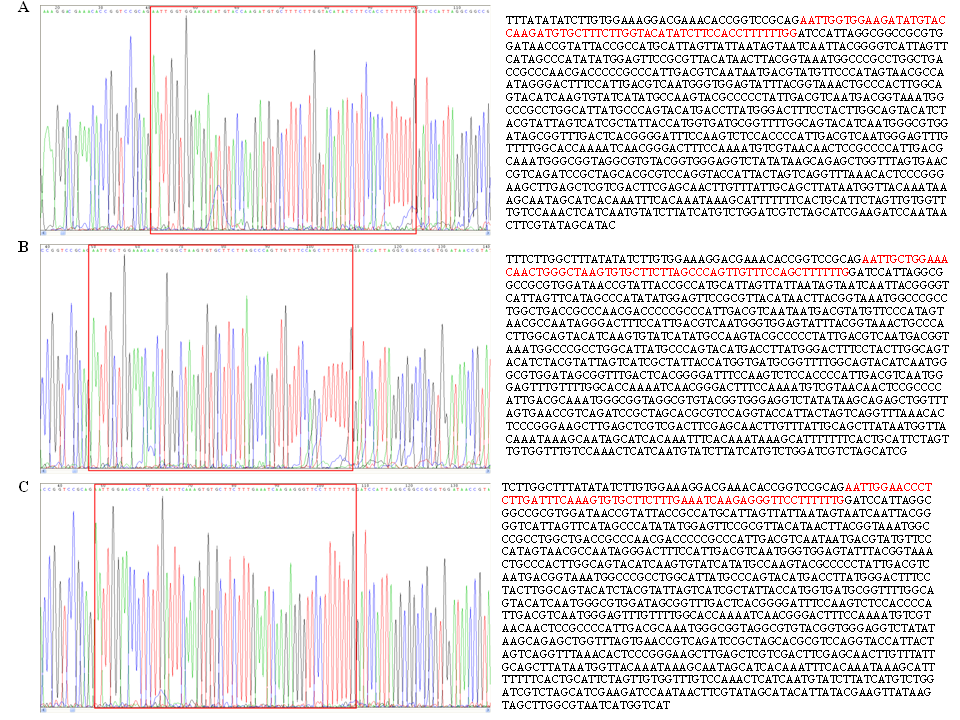
**

# Fig. S2. Three different shRNA-β-tubulin sequences. (A) β-tubulin shRNA 1 sequence, (B) β-tubulin shRNA 2 sequence, (C) β-tubulin shRNA 3 sequence.
